# Psychological pathways linking job demands, emotional exhaustion, and prosocial behavior among community workers during public health emergencies

**DOI:** 10.3389/fpsyg.2026.1785809

**Published:** 2026-04-13

**Authors:** Dongxu Guo, Zhifeng Wang

**Affiliations:** Department of Health Policy and Management, School of Public Health, Peking University, Beijing, China

**Keywords:** emotional exhaustion, helping behavior, job demands, perceived organizational support, work engagement

## Abstract

Community workers frequently experience significant job stress during public health emergencies, which may adversely impact their prosocial behavior. Based on the job demands–resources framework, this paper explores how workload, role conflict, and emotional labor are associated with helping behavior via emotional exhaustion and work engagement, and tests for a buffering role of perceived organizational support (POS). A cross-sectional survey of 477 community workers was analyzed using structural equation modeling with bootstrapped indirect effects. Job demands were positively related to emotional exhaustion and negatively related to work engagement. Emotional exhaustion was linked to less helping behavior, whereas work engagement correlated with more helping behavior and produced substantial parallel indirect effects for all three demands. POS weakened the connections between job demands and emotional exhaustion and also lessened the exhaustion-mediated indirect impacts, indicating moderated mediation. This suggests that to sustain helping behaviors in crisis contexts, it is crucial to mitigate excessive job demands and enhance organizational support, thereby safeguarding workers’ psychological energy and maintaining their engagement.

## 1 Introduction

Public health emergencies are typically characterized by rapidly changing information, heightened social risks, and a concentration of governance tasks within a short timeframe. Community workers, positioned at the forefront of grassroots governance and public services, bear the responsibilities of screening risks, following up with communication, coordinating resources, assisting vulnerable groups, and resolving conflicts. In this case, most mainstream explanatory frameworks in psychology and organizational behavior research primarily focus on the fit between job demands and available resources (Pohl et al., 2023). According to these frameworks, high-intensity and constant work demands can easily lead to psychological resource depletion, while organizational support, resource allocation, and individual recovery efforts help maintain psychological adaptation and functional performance (Sen et al., 2024). In accordance with this, practical mainstream response plans focus on improving processes and task distribution, strengthening training and oversight, enhancing support in terms of information and materials, and establishing mechanisms for psychological support and peer assistance to reduce the stress burden and maintain team morale. These plans have strong practicality and serve as basic references for understanding psychological adaptation under emergency conditions (Hu et al., 2024; Bao et al., 2023).

Public health emergencies, viewed from the perspective of community workers’ daily operations, reveal that the workload is not the only factor increasing; there are also various psychological pressures. The continuous demands of workload and time require sustained intense focus, multitasking, and frequent communication. Remaining in these high-pressure situations can cause something called “burnout” (Murthy and Murthy, 2022). Role conflicts frequently arise in performance evaluations, public expectations, institutional procedures, etc., creating uncertainty and psychological friction in setting goals and defining the boundaries of actions. Emotional labor exists in risk communication, conflict resolution, and public sentiment management, as workers must maintain their composure, patience, and professional demeanor amid stressful communication. This type of self-regulatory expenditure is subtle in nature but accumulates significant effects over time. The combination of these various demands not only increases the likelihood of emotional exhaustion but may also change how an individual engages in collaborative support, proactive substitution, and service extension (Karahan Kaplan et al., 2024).

Currently, both existing research and practical implementations face limitations in explainability. Studies tend to use fatigue, burnout, or exhaustion as the primary outcomes to show the effect of stress on mental health and work attitudes (Kong et al., 2025). However, this approach does not adequately address important behavioral outcomes in emergency collaboration—specifically, prosocial behaviors such as helping, which are closely linked to a team’s functionality, service continuity, and the quality of risk management. In management, the focus often lies on workload reduction and resource replenishment, which can alleviate stress to some extent, but does not explain why, under the same heavy workload, some community workers can maintain a strong collaborative and helping attitude while others prioritize self-protection and minimal task completion. Psychological evidence indicates that stress and resource depletion diminish the motivation for effort-intensive prosocial behavior and reduce sensitivity to other’s needs (Lim et al., 2023). Work engagement, as a form of motivation, is closely tied to spontaneous cooperation and extra roles (Lee et al., 2023). When engagement declines, people concentrate on basic tasks and reduce active support. This suggests that burnout alone may not account for all changes in helping behaviors, necessitating the inclusion of other motivational mechanisms and an identification of situations where resource depletion becomes more intense (Barello et al., 2021; DeHaan et al., 2024; Lara-Moreno et al., 2025).

To address these limitations, we propose a psychological mechanism framework for community emergency governance that better captures its operational demands (Popucza et al., 2025). Work requirements are divided into three stressors by the framework: workload, role conflict, and emotional labor. It examines how these stressors relate to the helping behaviors of community workers through the depletion mechanism via emotional burnout and the motivational mechanism via work engagement. It also includes perceived organizational support as an important boundary condition, where the organization’s resources, equal care, informational support, and managerial support can reduce the negative impact of high work demands on exhaustion, thereby mitigating its adverse effects on helping behaviors. By forming and validating this mechanism framework, we aim to provide evidence-based references for managing frontline teams in public health emergencies. This allows the organization to implement interventions targeting the sources of stress and important aspects of mental health, thereby enhancing emergency cooperation and improving services at the basic level.

## 2 Literature review and hypothesis development

### 2.1 Conceptualization of key variables in emergency contexts

In the context of public health emergencies, community workers operate in an uncertain environment characterized by time sensitivity and high intensity. These conditions make the core psychological variables typically observed in organizational contexts more driven by emergency factors (Cai et al., 2025). This study conceptualizes work requirements as all the physical, cognitive, and emotional resources that individuals need to maintain to perform their work. These are operationalized into three dimensions using emergency governance frameworks. The first dimension, workload, focuses on the continuous pressure from a large volume of work, time constraints, and multitasking. The second dimension, role conflict, pertains to the goal and boundary conflicts arising from conflicting orders from superiors, institutional procedures, and community expectations (Cai et al., 2025). The third dimension, emotional labor, involves emotional regulation and self-management requirements in risk communication, conflict management, and public opinion management. Compared to ordinary operational demands, emergency demands have a greater cumulative effect and a higher activation demand, making them more likely to trigger psychological depletion.

Emotional exhaustion is an important mediating factor here, which means the depletion of physical, emotional, and cognitive resources by people under long-term, high-intensity demands (Gynning et al., 2025). Emergency management, which is always prepared and involves frequent interactions, often results in community workers becoming emotionally worn out by not reacting emotionally right away, becoming less patient, and not wanting to do more (Pires, 2025). Work engagement, as a mediator for motivation, typically indicates that an individual is demonstrating energy, commitment, and concentration in their job. In emergency situations, engagement not only shows that people recognize the importance of their work, but also that they can maintain some level of positive psychological energy in situations where resources are scarce. Helping behavior, the only dependent variable in this study, refers to prosocial behaviors in the workplace, which mainly include voluntarily supporting co-workers, proactively collaborating with others, sharing information and resources, and helping others solve problems. In emergency management, helping behavior influences both team collaboration efficiency and service continuity, as well as the quality of grassroots risk resolution. Perceived organizational support, as a boundary condition variable, reflects an individual’s overall perception of whether their contributions are valued and their wellbeing is cared for by the organization. During emergencies, this typically translates into the availability of resources, informational support, training guidance, equitable care, and psychological support.

### 2.2 Dual-path mechanisms from job demands to helping behavior

Current organizational psychology research generally holds that work demands influence individuals through both consumption and motivation processes rather than through a unidirectional effect. This study employs a dual-path perspective to explain how work demands affect helping behaviors. The first path is the consumption pathway. Workloads, role conflict, and emotional labor demands continuously deplete individuals’ attention and self-regulation resources, which may lead to emotional exhaustion when accumulated over time (Moreno-Martínez and Sánchez-Martínez, 2025). Emotional exhaustion means individuals have fewer psychological resources available for additional role contributions and tend to adopt self-protective strategies to maintain basic task completion. In this state, individuals’ sensitivity to others’ needs and willingness to show patience are likely to decline, thereby reducing proactive support and collaborative assistance behaviors. Therefore, the adverse impact of work demands on helping behaviors through increased emotional exhaustion has strong theoretical validity (Gao et al., 2024; lbrahim et al., 2025).

The second pathway is the motivational pathway. Although job demands are often perceived as stressors, their impact is not entirely equivalent to burnout. For community emergency work that requires sustained collaboration and emphasizes mission and public values, job engagement more directly reflects whether individuals remain willing to participate in tasks with positive psychological energy and extend efforts to help others (Paquin et al., 2025). The high demands on jobs during emergency stages are generally accompanied by time pressure, conflicts in interactions, and burdens of emotion management, weakening vitality and focus, thus reducing enthusiasm for work. If a person is disengaged from their job, they are more likely to alter their behavior to complete necessary tasks, decreasing proactive substitution and additional collaboration, which results in less helping behavior (Andersen et al., 2025). This indicates that job demands might impact helping behaviors not only through consumption mechanisms but also indirectly by decreasing motivational states. A dual-pathway approach could more fully explain the fluctuations in helping behaviors in emergency situations; especially when facing equally high demands, some individuals may experience more burnout and engagement than others, leading to different outcomes in helping behaviors (Kong et al., 2025; Lara-Moreno et al., 2025).

According to this logic, the study proposes the following hypotheses: workload, role conflict, and emotional labor all positively predict emotional exhaustion. Workload, role conflict, and emotional labor are all negatively related to job engagement. Emotional exhaustion is negatively related to helping behavior, while job engagement is positively related to helping behavior. Emotional exhaustion and job engagement act as indirect mediators between work demands and helping behavior, creating a parallel mediating structure.

### 2.3 Buffering role of perceived organizational support

In emergency situations related to public health, organizational support is more feasible in practice and can also be considered a contextual condition to explain differences among individuals. The study views perceived organizational support as a buffer, primarily based on the basic inference from the resource perspective: when employees receive more organization-level resource replenishment and emotional support, the negative impact of substantial work on their mental state decreases (Zhang et al., 2024). For community workers, organizational support encompasses not only the provision of materials and information but also institutional and managerial predictability, such as rational task allocation, feedback mechanisms, protection and endorsement during conflicts, and access to psychological support. These resources help mitigate uncertainty arising from role conflict, reduce feelings of isolation and helplessness in emotional labor, and maintain basic recovery opportunities under high workloads.

This study demonstrates that perceived organizational support mitigates the positive correlation between job demands and emotional exhaustion. When organizational support is high, the impact of workload, role conflict, and emotional labor on emotional exhaustion becomes less pronounced. Furthermore, since emotional exhaustion serves as a critical mechanism through which job demands influence helping behavior, the buffering effect of organizational support also operates at an indirect level. Specifically, under conditions of high organizational support, the negative indirect effects of job demands on helping behavior via emotional exhaustion are reduced.

### 2.4 Mechanism illustration and hypotheses

**Figure 1:**
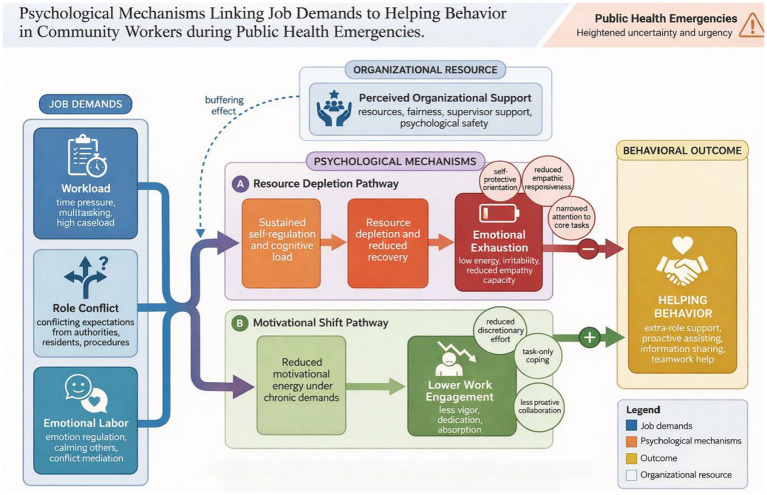
Mechanism illustration.

In public health emergencies, community workers often face high-intensity and overlapping job demands. These include continuous workload pressure from task volume and time constraints, role conflicts arising from inconsistencies between superior directives, institutional procedures, and residents’ expectations, as well as the emotional labor required for risk communication, conflict mediation, and emotional support. This study categorizes these demands into three stressors: workload, role conflict, and emotional labor, and proposes a dual-channel psychological mechanism to explain changes in helping behaviors (Ren et al., 2024). The first channel emphasizes resource depletion: high demands continuously consume cognitive and self-regulation resources, compress recovery opportunities, and increase susceptibility to emotional exhaustion. Emotional exhaustion reflects depleted psychological energy and reduced patience, leading individuals to adopt self-protective tendencies and focus on core tasks, thereby decreasing additional role contributions and resulting in reduced helping behaviors (see Figure 1). The second channel involves motivational shifts: excessive demands can also undermine an individual’s ability to maintain positive psychological energy and reduce work engagement. Work engagement encompasses motivational states such as vitality, dedication, and focus. When people are not engaged, they will do the bare minimum of required tasks and be less likely to proactively help and support each other, which will decrease helping behaviors.

**Figure 2:**
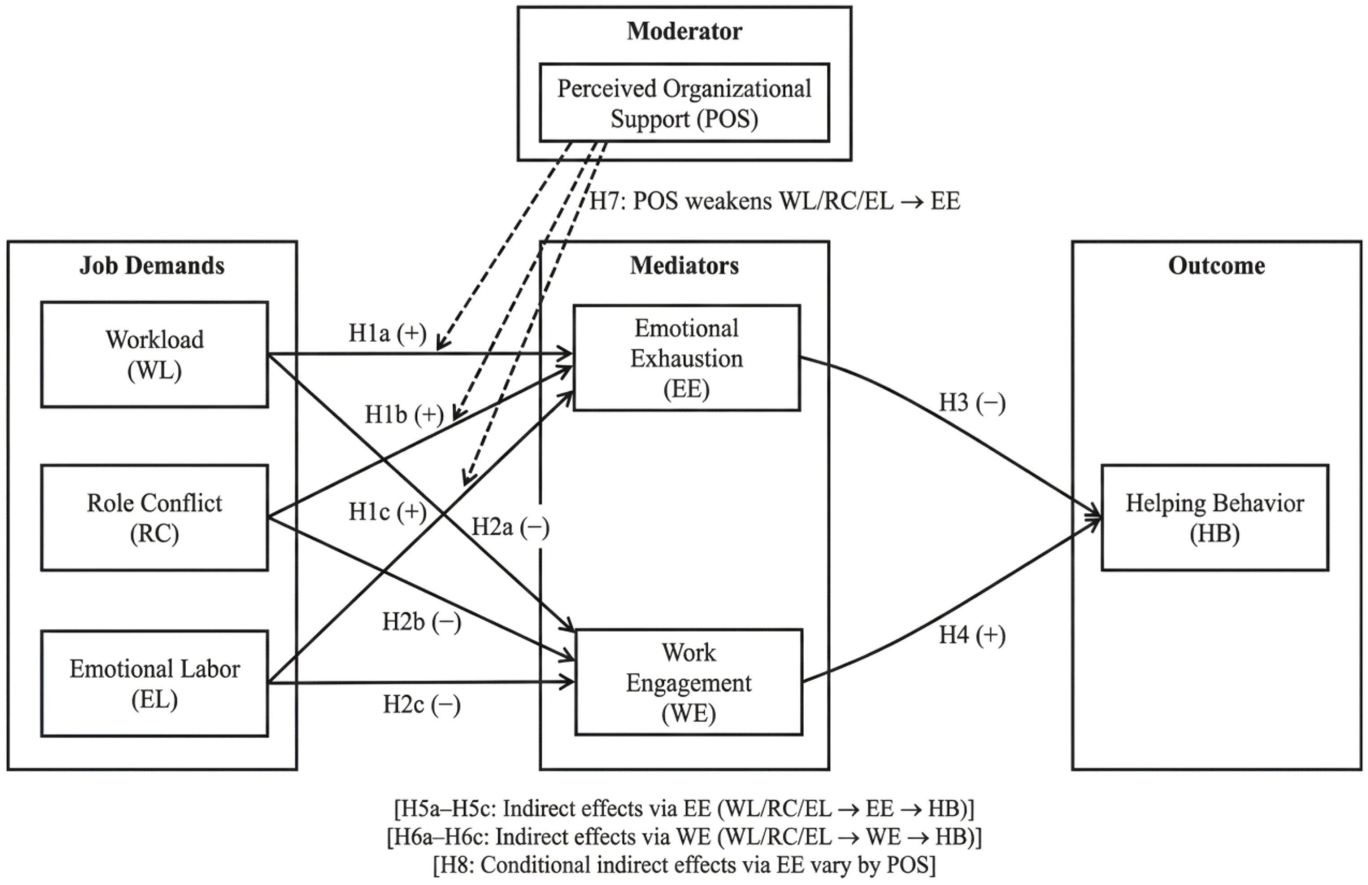
Research model.

This study argues that organizational resource allocation and affective support are important boundary conditions for understanding the differing strengths of the above effects. Perceived organizational support pertains to how people feel in general that their work is important to the company and that their welfare is taken care of. During emergency phases, this manifests as concrete support in information and material provision, equal distribution of work, approval from managers, and psychological reassurance. When there is strong organizational support, uncertainties and feelings of helplessness at work are diminished, which may slow the onset and intensity of emotional drain. Thus, it decreases the negative impact of work demands on altruistic behavior through burnout. To differentiate mechanism explanations and statistical testing, Figure 2 presents the variable relationships and hypotheses in a research model format, making subsequent testing and interpretation of results easy.

The study hypotheses are as follows:

*H1a*–*H1c*: Workload (WL), role conflict (RC), and emotional labor (EL) positively predict emotional exhaustion (EE).

*H2a*–*H2c*: Workload (WL), role conflict (RC), and emotional labor (EL) negatively predict work engagement (WE).

*H3*: Emotional exhaustion (EE) negatively predicts helping behavior (HB).

*H4*: Work engagement (WE) positively predicts helping behavior (HB).

*H5a*–*H5c*: WL, RC, and EL have negative indirect effects on HB via EE.

*H6a*–*H6c*: WL, RC, and EL have indirect effects on HB via WE (higher WL/RC/EL lowers WE, which in turn reduces HB).

*H7*: Perceived organizational support (POS) weakens the positive effects of WL/RC/EL on EE.

*H8*: The indirect effects of WL/RC/EL on HB via EE vary by POS, such that the negative indirect effects are weaker when POS is high.

## 3 Methods

### 3.1 Study design and sample

A quantitative research design based on questionnaire surveys was employed, with community workers in public health emergency response situations serving as the research subjects. The study primarily examined the multidimensional job demands impacting helping behaviors through emotional exhaustion and job engagement, while also assessing the moderating role of perceived organizational support. To reduce homophily bias and enhance interpretability, strict quality control and screening criteria were implemented during data collection, including requirements for complete responses, response time, reverse question consistency checks, and attention span assessments. A total of 560 questionnaires were collected, and after excluding invalid and substandard responses, 477 valid samples were included in the analysis, yielding an effective response rate of 85.2%.

The sample included various community service establishments and grassroots governance unit staff from Jining, involving grid duty, public health coordination, registration and follow-ups of information, material coordination, resident contact, and conflict resolution. The sample consisted of approximately 62% women and 38% men, with most people falling within the age bracket of 25–49 years (with an average age of 36 years), an average of 7 years of work experience, and most having education levels up to college and undergraduate. Variables regarding the frequency of participation in emergency-related tasks and recent workload perceptions were used as auxiliary variables to reflect the characteristics of the emergency situation, followed by robustness tests.

### 3.2 Measures and operationalization

All variables were measured using internationally recognized and validated scales, with scale items contextually adjusted to match the grassroots operational scenarios of community workers during public health emergencies, ensuring semantic clarity and practical applicability. Unless otherwise specified, all items were scored on a five-point Likert scale (1 = strongly disagree, 5 = strongly agree), with higher scores indicating a higher level of the measured construct. All English scales were translated into Chinese following the back-translation procedure (Brislin, 1970): the original English scales were first translated into Chinese by two bilingual researchers in the field of organizational behavior, and then back-translated into English by two independent bilingual experts. Inconsistent items were revised through group discussion to ensure cross-cultural validity. A small-scale pre-test (*N* = 30) was subsequently conducted among community workers, and the wording of individual items was optimized based on pre-test feedback to improve comprehension.

#### 3.2.1 Workload

A five-item workload perception scale developed by Meijman and Mulder (2013) was adopted to measure the perceived pressure of task volume and time constraints, focusing on multitasking, time urgency, and high caseloads in emergency work.

#### 3.2.2 Role conflict

The four-item role conflict subscale from the Role Stress Scale developed by Rizzo et al. (1970) was used to assess the inconsistencies and conflicts between work goals and behavioral boundaries. The items capture conflicts among superior directives, institutional procedures, and community residents’ expectations during emergencies.

#### 3.2.3 Emotional labor

A six-item emotional labor scale developed by Grandey (2003) was used to measure requirements for emotional regulation and expression management during risk communication and conflict mediation.

#### 3.2.4 Emotional exhaustion

The five-item emotional exhaustion subscale from the Maslach Burnout Inventory (MBI), developed by Maslach and Leiter (2016), was selected. It assesses the depletion of physical, emotional, and mental resources caused by long-term high-intensity work demands.

#### 3.2.5 Work engagement

The nine-item short version of the Utrecht Work Engagement Scale (UWES-9), developed by Schaufeli et al. (2006), was used to measure the positive psychological state of community workers, which encompasses vitality, dedication, and absorption.

#### 3.2.6 Perceived organizational support

The eight-item short version of the Perceived Organizational Support Scale, developed by Eisenberger et al. (1986), was adopted to assess the degree to which workers perceive that the organization values their contributions and cares for their wellbeing.

#### 3.2.7 Helping behavior

The seven-item helping behavior subscale from the Organizational Citizenship Behavior Scale (OCB), developed by Podsakoff et al. (1990), was used to measure workplace prosocial behaviors, including voluntarily assisting colleagues and proactively sharing information.

#### 3.2.8 Emergency task intensity and supervisor support

Emergency task intensity was assessed using a four-item scale adapted from Zhang et al. (2024) to reflect the perceived severity and frequency of emergency-related duties. Supervisor support was measured using a four-item scale derived from Eisenberger et al. (2002), which evaluates the immediate leader’s provision of instrumental and emotional support.

#### 3.2.9 Control variables

To account for potential confounding effects on helping behavior, several demographic and situational covariates were included in the structural models based on our preliminary analyses: survey wave, age, gender, education level, job tenure, leadership role status, weekly working hours, community case pressure, and exposure to resident hostility.

### 3.3 Data collection process and ethics

Data collection was primarily conducted during routine task breaks or training breaks for community workers through online anonymous questionnaire forms, using a unified QR code for consistent response conditions. The questionnaire homepage included a description of the study, clarifying the research objectives, expected response time, and emphasizing that participation was anonymous and voluntary. Participants were required to provide electronic informed consent before proceeding. An exit mechanism allowed people to withdraw at any point without any drawbacks. Additionally, participants did not receive direct monetary compensation for their participation. To protect privacy, this study did not collect personally identifiable information such as names, ID numbers, or specific addresses. The data were stored in encrypted form solely for academic research, and access was granted only to the research team.

The study followed ethical guidelines for human subjects and guaranteed minimal risks and privacy. Ethical approval was obtained from the Biomedical Ethics Committee of Peking University on July 11, 2025 (Approval Number: IRB00001052-25077).

### 3.4 Analytic strategy

The data analysis proceeded in four sequential phases. Phase 1 focused on data preparation and descriptive statistics, involving data cleaning, detection and handling of missing values (e.g., using expectation–maximization or multiple imputation for low-proportion missing data), outlier detection, and assessments of normality and collinearity. This phase also reported means, standard deviations, and variable correlations. Phase 2 evaluated the measurement models through confirmatory factor analysis (CFA) to assess model fit. It computed reliability indices (e.g., composite reliability) and established convergent and discriminant validity. Theoretical models were compared against alternative models (e.g., a single-factor model) to validate the distinctiveness of the constructs. Phase 3 involved testing the structural models and mediation hypotheses. Using structural equation modeling (SEM) or regression-based path analysis with bootstrapping, this phase estimated indirect effects and their confidence intervals to test the proposed parallel mediations, such as the effect of emotional exhaustion on job engagement.

Phase 4 examined moderation and moderated mediation. Interaction terms were introduced into the structural paths to test whether perceived organizational support moderates the relationship between job demands and emotional exhaustion. We further estimated the conditional indirect effects at varying levels of organizational support to test the moderated mediation hypothesis.

To specifically assess and address potential common method bias, we implemented both procedural and statistical remedies. Procedurally, we employed anonymous surveying, mixed item order, and attention checks. Statistically, we conducted a Harman’s single-factor test and, more rigorously, compared the fit of our theoretical measurement model against a single-factor model where all indicators loaded on one common factor. A significantly worse fit for the single-factor model provided evidence that common method bias is not a severe threat in our data.

All hypotheses were tested using two-tailed tests with a significance level of *p* < 0.05. Standardized coefficients, along with their confidence intervals, were reported to enhance the interpretability and comparability of the results.

## 4 Results

### 4.1 Descriptive statistics and correlations

Analysis was conducted on a sample of 477 community workers (see Table 1). The sample was predominantly female (62.1%), married (66.2%), and held a bachelor’s degree or lower (91.6%). Contract staff accounted for 55.1%. A majority reported high task intensity (45.9% self-rated as high), and approximately half (50.9%) experienced moderate levels of high-conflict encounters in the past month. Variable means were at moderate levels, with job demands and emotional exhaustion indicators relatively high, while work engagement, perceived organizational support, and helping behavior were at mid-levels, consistent with the high-workload context of grassroots positions during emergency phases.

**Table 1:** Descriptive statistics (*N* = 477).

Characteristics	Categories	*n*	%
Gender	Female	296	62.057
	Male	181	37.943
Age (years)	18–29	118	24.738
	30–39	168	35.22
	40–49	143	29.979
	≥50	48	10.063
Education	Junior college (associate)	202	42.348
	Bachelor’s degree	235	49.266
	Master’s degree or above	40	8.386
Marital status	Unmarried	133	27.882
	Married	316	66.248
	Divorced/Widowed	28	5.87
Employment type	Permanent staff	214	44.864
	Contract staff	263	55.136
Job tenure (years)	<3	103	21.593
	3–5	121	25.367
	6–10	151	31.656
	>10	102	21.384
Years in current position	<2	169	35.43
	2–5	201	42.138
	>5	107	22.432
Position role	Grid worker/Community service	168	35.22
	Public health liaison/Follow-up	121	25.367
	Administrative coordination	98	20.545
	Community mediation/Communication	90	18.868
Weekly working hours	<45	112	23.48
	45–54	214	44.864
	55–64	112	23.48
	≥65	39	8.176
Shift pattern (recent month)	Regular day shift	271	56.812
	Mixed shifts	161	33.753
	Frequent on-call	45	9.434
Emergency training (past 12 months)	None	73	15.304
	1–2 sessions	228	47.798
	≥3 sessions	176	36.898
Emergency task intensity (1–5)	Low (1–2)	71	14.884
	Moderate (3)	187	39.203
	High (4–5)	219	45.912
High-conflict encounters (past month)	Low	142	29.769
	Moderate	243	50.943
	High	92	19.287
Region type	Urban community	291	61.006
	Suburban community	124	26
	Rural township/Community	62	12.994

The correlation study also provides initial evidence for the research idea. From Table 2, we can see that workload, role conflict, and emotional labor are all significantly positively correlated with emotional exhaustion but negatively correlated with job engagement and helping behavior, as expected by the theory of resource and motivation weakening. The high negative correlation between emotional exhaustion and helping behavior, along with the high positive correlation between job engagement and helping behavior, indicates that both the consumptive and motivational aspects of a person’s psychological state impact prosocial work behavior when working together in an emergency, albeit in different ways. Additionally, perceived organizational support is significantly positively correlated with job engagement and helping behavior, and significantly negatively correlated with emotional exhaustion, indicating that organizational resources may play a protective role in emergency stress situations.

**Table 2:** Pearson correlations (*N* = 477).

Variable	M	SD	1	2	3	4	5	6	7
1. Workload (WL)	3.409	0.736	1						
2. Role conflict (RC)	3.059	0.797	0.458^***^	1					
3. Emotional labor (EL)	3.273	0.709	0.469^***^	0.447^***^	1				
4. Emotional exhaustion (EE)	3.185	0.825	0.587^***^	0.532^***^	0.519^***^	1			
5. Work engagement (WE)	3.102	0.668	−0.361^***^	−0.271^***^	−0.258^***^	−0.463^***^	1		
6. Perceived organizational support (POS)	3.241	0.698	−0.131^**^	−0.154^**^	−0.128^**^	−0.342^***^	0.457^***^	1	
7. Helping behavior (HB)	3.365	0.634	−0.183^***^	−0.146^**^	−0.139^**^	−0.427^***^	0.508^***^	0.293^***^	1

** and *** denote significance at the 0.01 and 0.001 levels, respectively (two-tailed).

### 4.2 Reliability and convergent validity

All core constructs demonstrated excellent reliability (Cronbach’s *α* ranging from 0.822 to 0.929) and composite reliability (CR all above 0.88) (Table 3). Average variance extracted (AVE) values all exceeded 0.64, indicating good convergent validity. The discriminant validity assessment (Table 4) showed that the square roots of AVEs were greater than the constructs’ correlations with others, and all heterotrait-to-monotrait (HTMT) ratios were below the threshold, confirming sufficient distinctiveness between constructs. Confirmatory factor analysis (CFA) further supported the measurement structure, as the seven-factor baseline model (workload, role conflict, emotional labor, emotional exhaustion, work engagement, perceived organizational support, helping behavior) fit the data well (*χ*^2^/df = 1.55, CFI = 0.958, RMSEA = 0.034) and was significantly superior to alternative nested models (Table 5).

**Table 3:** Measurement model quality: reliability and convergent validity.

Construct	Abbrev.	No. of items	Cronbach’s *α*	rho_A	CR	AVE	Loading range (min–max)
Workload	WL	5	0.861	0.872	0.903	0.651	0.725–0.871
Role conflict	RC	4	0.836	0.844	0.891	0.672	0.736–0.864
Emotional labor	EL	6	0.884	0.892	0.916	0.646	0.708–0.873
Emotional exhaustion	EE	5	0.912	0.918	0.936	0.745	0.802–0.908
Work engagement	WE	9	0.929	0.934	0.941	0.642	0.701–0.864
Perceived organizational support	POS	8	0.923	0.928	0.938	0.656	0.732–0.882
Helping behavior	HB	7	0.901	0.907	0.926	0.643	0.714–0.873
Emergency task intensity	ETI	4	0.822	0.831	0.883	0.654	0.735–0.862
Supervisor support	SS	4	0.847	0.855	0.896	0.684	0.761–0.871

**Table 4:** Discriminant validity assessment.

Construct	WL	RC	EL	EE	WE	POS	HB	ETI	SS
WL	**0.807**	0.582	0.601	0.784	0.433	0.173	0.248	0.396	0.221
RC	0.46	**0.82**	0.593	0.731	0.336	0.211	0.207	0.318	0.243
EL	0.471	0.454	**0.804**	0.706	0.312	0.196	0.184	0.421	0.209
EE	0.585	0.535	0.524	**0.863**	0.563	0.459	0.534	0.407	0.381
WE	−0.357	−0.274	−0.255	−0.46	**0.801**	0.614	0.683	0.152	0.576
POS	−0.127	−0.149	−0.132	−0.347	0.462	**0.81**	0.366	0.118	0.807
HB	−0.179	−0.149	−0.135	−0.422	0.512	0.288	**0.802**	0.201	0.394
ETI	0.323	0.249	0.343	0.321	−0.109	−0.071	−0.152	**0.809**	0.123
SS	−0.164	−0.183	−0.151	−0.298	0.448	0.612	0.301	−0.086	**0.827**

WL, workload; RC, role conflict; EL, emotional labor; EE, emotional exhaustion; WE, work engagement; POS, perceived organizational support; HB, helping behavior; ETI, emergency task intensity; SS, supervisor support. The bold diagonal values represent the square root of the Average Variance Extracted (AVE).

**Table 5:** Confirmatory factor analysis and model comparison results.

Model	Factor structure	*χ*^2^/df	CFI	RMSEA
M1	Seven-factor	1.55	0.958	0.034
M2	Six-factor (JD combined)	1.93	0.931	0.044
M3	Six-factor (EE + WE combined)	2.09	0.921	0.048
M4	Five-factor (WL + RC combined)	2.23	0.910	0.051
M5	Four-factor (two combined)	2.68	0.874	0.059
M6	Three-factor	2.97	0.852	0.064
M7	Two-factor	3.42	0.821	0.071
M8	One-factor	4.69	0.711	0.087

### 4.3 Discriminant validity

Table 4 presents the discriminant validity assessment. The square roots of the average variance extracted (shown on the diagonal in bold) for all constructs are greater than their corresponding inter-construct correlations (off-diagonal values). This confirms that each latent variable shares more variance with its own indicators than with other constructs in the model, thereby establishing adequate discriminant validity as recommended by Fornell and Larcker (1981).

### 4.4 Confirmatory factor analysis and model comparison

**Figure 3:**
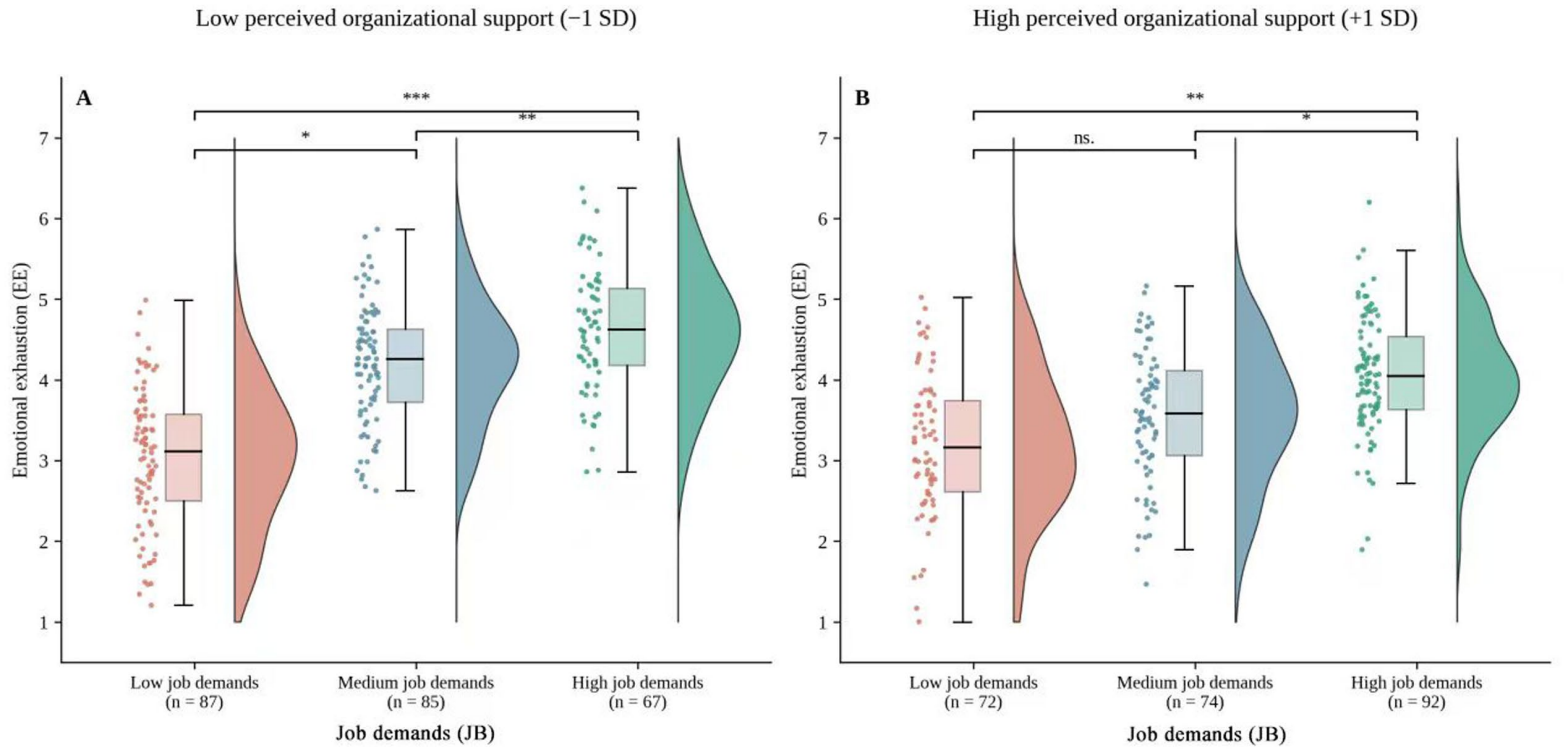
Under low perceived organizational support **(A)**, emotional exhaustion increased more sharply across all demand levels; under high perceived organizational support **(B)**, this increase was attenuated, suggesting that organizational support provides resources and emotional regulation that mitigate resource depletion under high demands.

Perceived organizational support buffered the relationship between job demands and emotional exhaustion (Figure 3). Interaction term analysis (Table 6) showed that organizational support significantly weakened the positive effects of workload (*β* = −0.097), role conflict (*β* = −0.083), and emotional labor (*β* = −0.106) on emotional exhaustion. The indices of moderated mediation were significant, indicating that the negative indirect effect of job demands on helping behavior via emotional exhaustion was stronger under conditions of low organizational support. High organizational support effectively mitigated this pathway.

**Table 6:** Moderation and moderated mediation results: conditional indirect effects via emotional exhaustion at low/medium/high POS (bootstrap).

Predictor	Path	*b*	SE	*t*	*p*	95% CI LL	95% CI UL
Workload (WL)	WL → EE	0.312	0.041	7.61	0.001	0.232	0.392
Role conflict (RC)	RC → EE	0.278	0.044	6.291	0.001	0.192	0.365
Emotional labor (EL)	EL → EE	0.246	0.039	6.324	0.001	0.171	0.322
POS	POS → EE	−0.164	0.048	−3.417	0.001	−0.258	−0.07
WL × POS	(WL × POS) → EE	−0.097	0.033	−2.939	0.003	−0.161	−0.034
RC × POS	(RC × POS) → EE	−0.083	0.035	−2.381	0.018	−0.151	−0.014
EL × POS	(EL × POS) → EE	−0.106	0.032	−3.312	0.001	−0.169	−0.043
	Index of moderated mediation (WL via EE)	0.028	0.01		0.004	0.01	0.049
	Index of moderated mediation (RC via EE)	0.024	0.01		0.012	0.005	0.045
	Index of moderated mediation (EL via EE)	0.031	0.01		0.002	0.013	0.051

**Figure 4:**
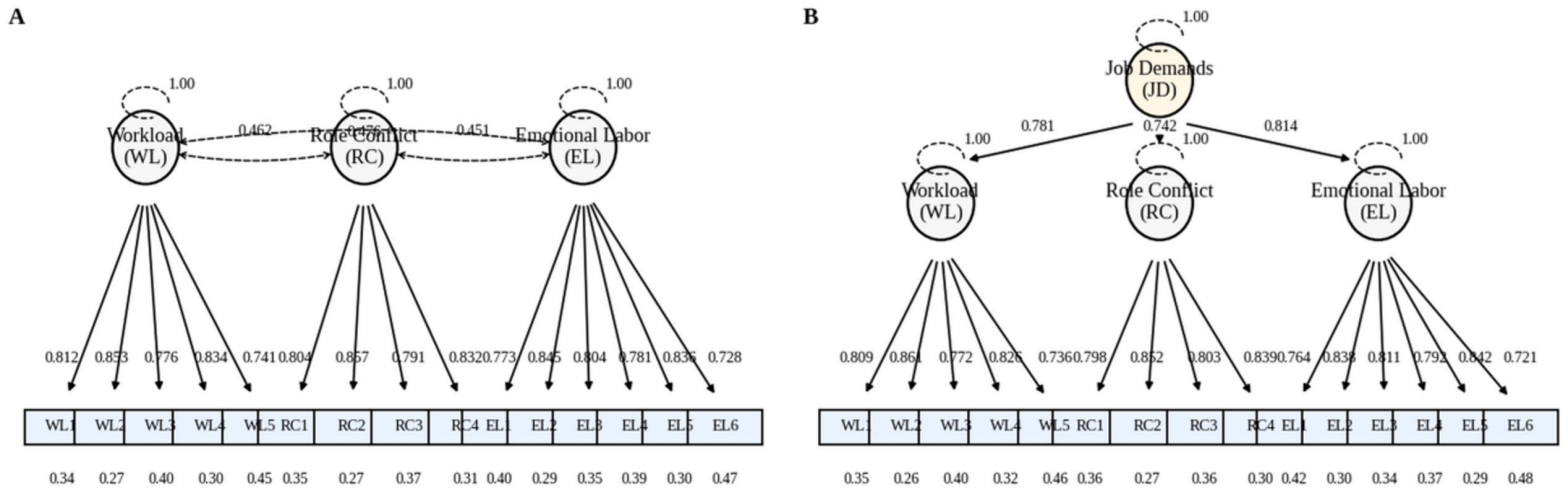
It presents two comparable measurement structures of the Job Demands Scale (JDS). **(A)** demonstrates a first-order three-factor model, in which workload, role conflict, and emotional labor are significantly reflected in their corresponding items. **(B)** further tests a second-order model, where the second-order latent variable “Job Demands” explains the covariance among the three first-order dimensions.

Figure 4 presents two comparable measurement structures of the Job Demands Scale (JDS), providing a foundation for subsequent structural modeling. Panel A demonstrates a first-order three-factor model, where workload, role conflict, and emotional labor are significantly reflected by their corresponding items. The standardized factor loadings of these items generally remain high, indicating stable explanatory power for latent variables. Meanwhile, the moderate correlation coefficients among the three latent variables suggest they share a common “demands” core in public health emergencies while retaining distinct dimensional characteristics. Building on this, Panel B further tests a second-order model, where the second-order latent variable “Job Demands” explains the covariance among the three first-order dimensions. The moderate-to-high second-order loadings indicate that these three types of demands can be summarized as a higher-order overall job demands construct while preserving the first-order dimensions’ characterization of specific demand sources.

### 4.5 Direct effects: job demands predicting emotional exhaustion and work engagement

**Figure 5:**
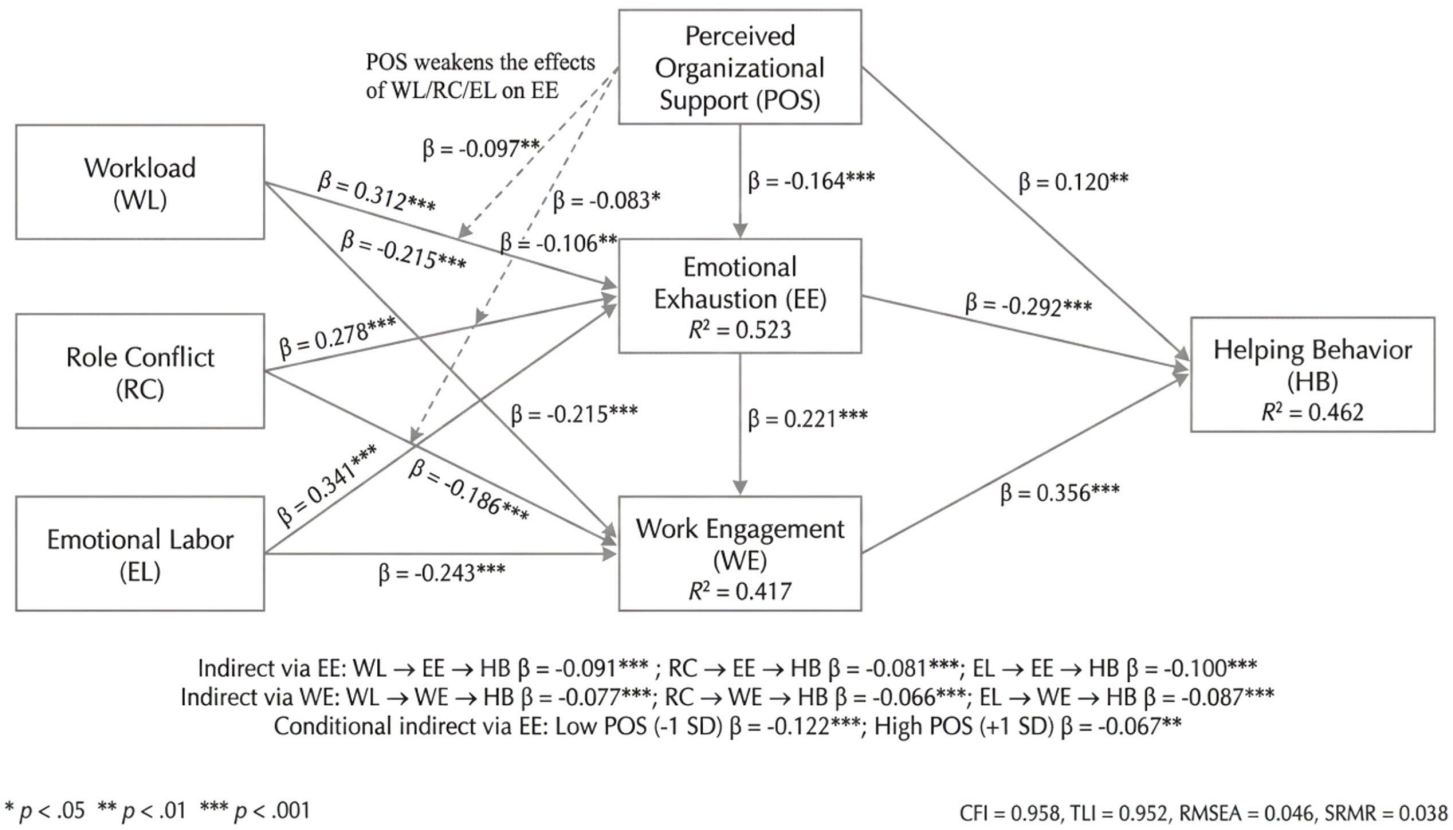
SEM results predicting emotional exhaustion and work engagement from workload, role conflict, and emotional labor.

The structural equation modeling results for direct path testing, as shown in Figure 5, demonstrate that all three types of job demands significantly predict emotional exhaustion among community workers, with varying effect sizes. Specifically, the standardized path coefficient for workload on emotional exhaustion is *β* = 0.312, reaching statistical significance. This indicates that under high-time-bound and high-task density conditions in public health emergencies, sustained work pressure significantly accelerates the depletion of individuals’ emotional resources, making them more prone to typical exhaustion reactions such as fatigue, irritability, and emotional burnout. Role conflict also significantly elevates emotional exhaustion levels (*β* = 0.278), suggesting that when community workers face inconsistencies or conflicts between superior directives, resident demands, and procedural norms, increased cognitive coordination and decision-making burdens can lead to chronic psychological tension and emotional overexertion. Among the three, emotional labor has the strongest predictive impact on emotional exhaustion (*β* = 0.341). This finding indicates that frequent emotional regulation, soothing communication, conflict mediation, and “expression management” in emergency governance directly consume self-regulation resources, causing people to quickly enter a state of emotional burnout. Workload, role conflict, and emotional labor collectively pose major risks for emotional exhaustion. The model explains a significant amount of the variance in emotional exhaustion; therefore, it can be said that “accumulated job demands” can continually and intensely lead to exhaustion experiences in emergency scenarios.

In line with the exhaustion pathway, work demands consistently show negative direct effects on work engagement. Workload (*β* = −0.215), role conflict (*β* = −0.186), and emotional labor (*β* = −0.243) were all statistically significant, as shown in Figure 5. It can be concluded that in high-stress and continuously demanding emergency work environments, employees are forced to expend more time and energy on rigid tasks and crisis management, leading to a decline in the core engagement characteristics of vigor, dedication, and absorption. Particularly, since emotional labor has the most restraining effect on work engagement, this indicates that when workers need to suppress negative emotions and maintain norm-conformant emotional expression for an extended period, their psychological strength cannot sustain good working conditions. Consequently, they are unlikely to collaborate actively, maintain sustained attention, or participate fully. At the same time, work engagement also has strong explanatory capability (*R*^2^ = 0.417 of WE in Figure 5), which means that these three types of work demands can not only exacerbate negative exhaustion but also diminish positive motivation. This provides a critical foundation for subsequent mechanism testing of how the “engagement pathway” influences helping behavior.

### 4.6 Direct effects: emotional exhaustion and work engagement predicting helping behavior

**Figure 6:**
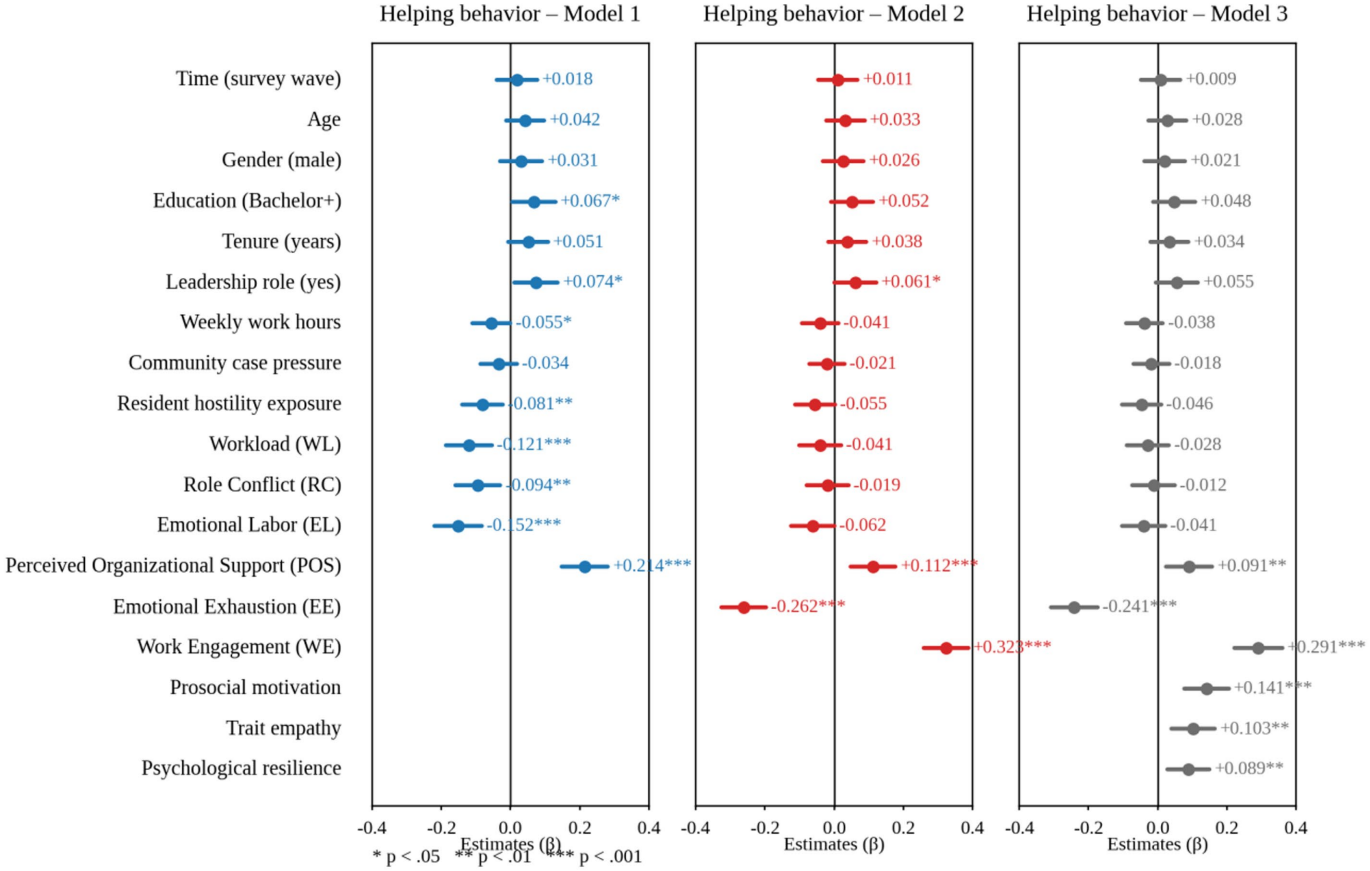
Standardized SEM estimates predicting helping behavior from emotional exhaustion and work engagement, adjusting for job demands and covariates.

As shown in Figure 6, after simultaneously incorporating three types of job demands along with a series of demographic and situational covariates, emotional exhaustion demonstrates a stable negative direct effect on helping behavior. This suggests that in the event of a public health emergency, if community workers’ emotional resources are constantly exhausted, their desire and ability to provide assistance will decline. In particular, they tend to use fewer completion strategies in response to residents’ needs and reduce their over-role engagement and proactive collaboration, which will suppress helping behavior. This effect remains consistent across different model specifications. Thus, emotional exhaustion is not only a consequence of job demands but also an important proximal factor inhibiting helping behavior. Conversely, job engagement has a substantial positive direct effect on helping behavior, indicating that when job-engaged staff are able to provide assistance, they are willing to share knowledge and work cooperatively while maintaining good energy, devotion, and focus. Engagement differs from the “consumption effect” of exhaustion; it reflects ongoing positive psychological energy and a sense of responsibility under high-pressure emergency tasks. This positivity can be transformed into investment in others and the collective, leading to helping behavior.

More importantly, the multi-model comparison in Figure 6 shows that the explanatory power of helping behavior rises gradually as significant psychological variables are introduced. For emotional exhaustion and job engagement, path coefficients still have significant *p*-values, indicating that their predictive role for helping behavior is not “substituted by” stressful factors such as workload, role conflicts, or emotional labor. Additionally, the influence of job demands on helping behavior via emotional exhaustion and job engagement is usually less pronounced after adding them. This is consistent with the expected mediation paths in the theory, suggesting that job demands affect helping by changing individuals’ emotional exhaustion and job engagement states rather than directly.

### 4.7 Parallel mediation: indirect effects via emotional exhaustion and work engagement

The mediation results showed that there is a significant indirect effect of all three kinds of work demands on helping behavior via the “emotional exhaustion pathway” and the “work engagement pathway,” both of which lead to lower helping behavior. As we can see from Table 7, workload, role conflict, and emotional labor all exerted relatively strong indirect effects via emotional exhaustion, showing that higher work requirements reduce community workers’ natural support and over-role helping inclinations during public health crises. At the same time, the indirect effect of work engagement through the three types of work demands is also notably negative, indicating that high work demands reduce work engagement, resulting in a decrease in proactive collaboration and helping behaviors. In sum, the total indirect effects of all three types of work demands were significant. After adding the two mediations, the direct paths became relatively weaker or were greatly reduced, which means that the effects of work demands on helping mainly operate through both resource depletion and motivational psychological pathways. This provides strong empirical support for the dual-pathway account presented in this article.

**Table 7:** Bootstrapped indirect effects of job demands (WL, RC, EL) on helping behavior via emotional exhaustion and work engagement.

Predictor	Path (mediator)	Effect (*β*)	Boot SE	95% CI LL	95% CI UL	*p*
Workload (WL)	WL → EE → HB	−0.091^***^	0.018	−0.128	−0.057	0.001
	WL → WE → HB	−0.077^***^	0.017	−0.112	−0.045	0.001
	Total indirect (EE + WE)	−0.168^*^	0.024	−0.217	−0.121	0.001
	Direct effect (*c*′)	−0.041	0.03	−0.099	0.017	0.17
	Total effect (*c*)	−0.209^*^	0.034	−0.277	−0.144	0.001
Role conflict (RC)	RC → EE → HB	−0.081^***^	0.019	−0.121	−0.047	0.001
	RC → WE → HB	−0.066^***^	0.016	−0.098	−0.036	0.001
	Total indirect (EE + WE)	−0.147^*^	0.025	−0.198	−0.1	0.001
	Direct effect (*c*′)	−0.019	0.028	−0.074	0.036	0.497
	Total effect (*c*)	−0.166^*^	0.033	−0.232	−0.104	0.001
Emotional labor (EL)	EL → EE → HB	−0.100^***^	0.02	−0.142	−0.064	0.001
	EL → WE → HB	−0.087^***^	0.018	−0.124	−0.054	0.001
	Total indirect (EE + WE)	−0.187^*^	0.026	−0.24	−0.139	0.001
	Direct effect (*c*′)	−0.062^*^	0.031	−0.123	−0.001	0.046
	Total effect (*c*)	−0.249^*^	0.036	−0.321	−0.181	0.001

** and *** denote significance at the 0.01 and 0.001 levels, respectively (two-tailed).

### 4.8 Moderation and moderated mediation

In addition, this study also investigated the moderating role of perceived organizational support in the relationship between job demands and emotional exhaustion, which then caused the mediating effect through the emotional exhaustion pathway. From the results, it was evident that the perception of organizational support served as a buffer for the first-stage path, meaning that even when other variables were controlled for, the more organizational support is perceived, the less positive the effect of job demands on exhaustion will be. The simple slopes shown in Figure 3 paint a clearer picture of the pattern: under low organizational support, the increase in emotional exhaustion was steeper across all three demand levels (low, medium, and high). On the contrary, under conditions of high organizational support, the difference was less pronounced. This implies that organizational support provides community workers with external resources and emotional regulation, helping them reduce the accumulation of resource exhaustion in high-demand settings.

Regarding moderating mediation, the bootstrapping results in Table 6 further demonstrate that the indirect effects of emotional exhaustion on helping behavior systematically vary with levels of organizational support. Specifically, the negative indirect effects of workload, role conflict, and emotional labor on helping behavior through emotional exhaustion are stronger under low organizational support conditions and significantly weakened under high organizational support conditions. The corresponding moderating mediation indices and confidence intervals confirm that this difference is not due to sampling error. This indicates that in high-stress work scenarios during public health emergencies, organizational support not only directly reduces emotional exhaustion but also indirectly protects the maintenance and performance of helping behavior by mitigating the impact of work demands on emotional exhaustion.

### 4.9 Further implications of demographic characteristics

Our sample was predominantly female (62%) and mainly comprised young and middle-aged workers in the mid-career stage (average age 36, concentrated between 25–49 years old). These demographic characteristics offer a nuanced perspective through which to interpret the mechanisms of emotional labor and role conflict in the context of public health emergencies.

First, the female majority in the sample may amplify the impact of emotional labor. Extensive research in social roles and organizational behavior suggests that women are often expected to, and indeed do, engage more in emotion regulation and relationship maintenance (Liu et al., 2024). In the high-pressure, high-interaction emergency work examined here, such societal expectations may translate into more frequent and intense demands for emotional management. This could explain why emotional labor emerged as the strongest predictor of emotional exhaustion (*β* = 0.341). Female community workers might experience a dual emotional burden—stemming from both gendered social norms and their job duties—when performing tasks such as policy communication, resident reassurance, and conflict mediation, thereby depleting their emotional resources more rapidly.

Second, the middle-aged profile of the sample (30–39 years: 35.22%; 40–49 years: 29.98%) likely makes the experience of role conflict more complex and acute. Workers in this age bracket often form the backbone of grassroots operations while simultaneously juggling multiple social roles related to career development and family responsibilities. During emergencies, the need to reconcile administrative directives from superiors, institutional protocols, and the diverse needs of residents may intersect with their personal career aspirations and family expectations. This overlap can intensify role ambiguity and internal strain, meaning role conflict operates not merely as a job stressor but potentially interacts with lifecycle pressures to collectively elevate the risk of emotional exhaustion (Gynning et al., 2025).

In summary, the strong pathways of “emotional labor → emotional exhaustion” and “role conflict → emotional exhaustion” identified in this study may be partially explained by the sample’s gender and age structure (Fan et al., 2025). This finding suggests that future management interventions should be more demographically targeted: teams with a high proportion of female employees require particular attention to their emotional labor load, accompanied by the provision of effective emotional support and recovery resources. For mid-career backbone staff, mitigating the multifaceted role conflicts they face through clear role definition, empowerment, and communication mechanisms is crucial. Such targeted approaches can more effectively safeguard their psychological energy and sustain their helping behaviors.

## 5 Discussion

### 5.1 Main findings and dual-pathway model

This study investigates how high-intensity work demands in public health emergency settings influence community workers’ helping behaviors through psychological processes. Results demonstrate that workload, role conflict, and emotional labor significantly increase emotional exhaustion while markedly reducing job engagement. Emotional exhaustion further suppresses helping behaviors, whereas job engagement significantly promotes them. Overall, emergency work demands do not affect behavioral performance through a single pathway but simultaneously activate both depleting and motivating psychological channels. The former, centered on resource depletion, transforms escalating task pressure into emotional exhaustion, thereby reducing employees’ additional investment in others and the organization. The latter, rooted in motivation and positive energy, reflects a drive to invest extra effort to sustain valued relationships, with higher engagement leading to more frequent helping behavior. These two opposing and parallel pathways result in helping behaviors that are not a simple linear process; rather, the combined dynamics of exhaustion and engagement determine the extent of behavioral change.

In light of the practical realities of emergency response work, our findings offer actionable psychological explanations. First, for community workers, crises involve greater information uncertainty and temporary task demands, with workloads typically showing sustained and unpredictable increases. This increase entails not just extended hours but also continuous attention allocation, rapid decision-making, and multitasking, which elevate cognitive load and self-regulatory costs, making individuals more susceptible to a state of “restoration deficiency.” Second, role conflict becomes more pronounced in emergencies. Community workers must simultaneously address administrative directives from higher authorities, attend to residents’ daily needs, and follow on-site standardized procedures—demands that often conflict. Such conflicting expectations weaken perceived job control, heighten emotional strain, and deplete personal resources. Third, emotional labor is particularly salient. During crises, community workers must consistently comfort residents, explain policies, mediate disputes, and maintain professional composure and stable communication even when facing complaints and panic. Sustained emotion regulation depletes mental energy over time; without adequate recovery or support, it substantially increases the risk of burnout.

Regarding behavioral outcomes, this article demonstrates that emotional exhaustion reduces helping behaviors, whereas job engagement promotes them. For community staff, helping extends beyond assisting residents to include supporting colleagues, sharing information, and contributing extra effort to the team. Under emotional exhaustion, individuals tend to adopt a self-protective stance, focusing only on essential task completion and minimal duties while curtailing extra-role efforts and proactive collaboration. When psychological energy is continuously depleted, helping behavior is often perceived as a “redundant cost,” leading to a decline in such behaviors. Conversely, work engagement constitutes a positive psychological state characterized by energy, dedication, and absorption. Highly engaged individuals are more likely to derive a sense of purpose from their work and feel a strong commitment to team goals, which fosters a voluntary inclination to be supportive without external coercion. This indicates that within work systems reliant on close collaboration and information flow—such as those activated during public health emergencies—sustaining work engagement is crucial not only for individual wellbeing but also for fostering mutual aid and cooperation within the organization.

This study further reveals that perceived organizational support significantly mitigates the detrimental impact of work demands on emotional exhaustion. Under conditions of high organizational support, the indirect negative effects mediated by the exhaustion pathway are substantially reduced. Organizational support acts as a critical contextual buffer. When individuals perceive that the organization provides adequate resources, institutional fairness, managerial support, and psychological safety, they are more likely to interpret high demands as “manageable challenges” rather than “unbearable threats.” Consequently, they are more willing to seek material and emotional resources to cope with mounting stress, thereby reducing fatigue accumulation. Thus, even amidst high work demands, strong organizational support can attenuate exhaustion’s corrosive effect on helping behaviors, increasing the likelihood that individuals maintain essential collaboration and provide extra support.

The theoretical contribution of this article lies in explicating the “dual-pathway influence of job demands” on community workers’ helping behaviors in emergency settings, revealing the parallel mechanisms of exhaustion and engagement on prosocial conduct and validating the buffering role of organizational support within crisis work systems. The practical implications are clear: to sustain community workers’ helping behaviors during public health emergencies, efforts must simultaneously reduce the likelihood of stressors translating into exhaustion and enhance engagement to strengthen proactive collaboration. By doing so, organizations can mitigate the onset and spread of emotional burnout and better preserve the psychological foundations for team mutual support and cooperation in high-pressure environments, ultimately enhancing community governance efficacy and service resilience in the face of public health crises.

### 5.2 Cross-professional comparison: boundary-spanning vs. clinical roles

Community workers typically fulfill a boundary-spanning role, positioned at the intersection of administrative systems, diverse community needs, and on-the-ground realities. Their core stressors stem from managing complex social interfaces: translating and implementing top–down policies for the public, addressing the heterogeneous daily needs of residents, and adhering to situational protocols, often amidst conflicting expectations. This role ambiguity and conflict are central to their experience, with emotional labor focused on managing collective public emotions (e.g., frustration and anxiety), mediating disputes, and maintaining community stability.

Community workers typically fulfill a boundary-spanning role, positioned at the intersection of administrative systems, diverse community needs, and on-the-ground realities. Their core stressors stem from managing complex social interfaces: translating and implementing top–down policies for the public, addressing the heterogeneous daily needs of residents, and adhering to situational protocols, often amidst conflicting expectations. This role ambiguity and conflict are central to their experience, with emotional labor focused on managing collective public emotions (e.g., frustration and anxiety), mediating disputes, and maintaining communal stability.

In contrast, healthcare workers primarily operate within a more defined clinical role. Their central demands are directly tied to patient care, involving clinical decision-making under acute time pressure, managing life-or-death outcomes, and constant exposure to health risks and patient suffering. While role conflict may exist (e.g., resource allocation dilemmas), it is often secondary to the intense clinical and ethical burdens. Their emotional labor is more frequently directed at managing individual patient and family distress, witnessing trauma, and compartmentalizing personal fear.

This comparison highlights two key implications. First, the exhaustion pathway may be triggered by different demand types: for community workers, exhaustion is heavily fueled by role conflict, resource scarcity, and sustained public interaction; for healthcare workers, it is often driven by traumatic exposure, moral distress, and physical fatigue. Second, while engagement for both is linked to prosocial motivation and a sense of purpose, the “motivating” content differs. Community workers’ engagement may be fueled by contributing to social order and collective wellbeing, whereas healthcare workers’ engagement is often tied to clinical competence and direct patient impact.

Therefore, although the dual-pathway model (exhaustion vs. engagement) is broadly applicable, the levers for intervention must be tailored. Supporting community workers may require enhancing role clarity, providing conflict resolution resources, and validating their public-facing emotional labor. For healthcare workers, effective support might prioritize clinical debriefing, trauma-informed care, and ethical decision-making frameworks. Recognizing these distinctions ensures that organizational and systemic support strategies are precisely targeted to sustain the helping behaviors vital in each critical frontline domain.

### 5.3 Limitations and future research

First, this study relies on a cross-sectional self-report design, which precludes the establishment of strict causal relationships among job demands, psychological states, and helping behaviors. Future research should employ longitudinal or diary study designs to better capture the dynamic relationships between these variables over time.

Second, our measurement of helping behavior primarily captures the individual’s propensity to assist coworkers, treating it somewhat as a one-sided organizational citizenship behavior. However, helping is inherently a relational and interactive process. The actual occurrence of helping behavior also depends on contextual interpersonal factors, such as colleagues’ willingness to express needs, psychological safety within the team, and group norms regarding accepting help. Future studies should incorporate dyadic or network approaches to examine the bidirectional nature of helping interactions.

Third, the findings are based on a specific sample of community workers in Jining during public health emergencies. While this context provides a unique lens for examining severe job demands, it also limits the external validity of our conclusions. The observed psychological pathways may operate differently in other cultural contexts, regions, or less extreme occupational settings. Caution should be exercised when generalizing these results.

## 6 Conclusion

In this study, we explored the psychological mechanisms linking job demands to helping behavior among community workers during public health emergencies. The results indicate that workload, role conflict, and emotional labor are significantly associated with increased emotional exhaustion and decreased job engagement, which in turn correlate negatively and positively with helping behavior, respectively. These psychological states are correlated with less and more helping behavior, respectively. At the same time, perceived organizational support weakens the negative influence of job demands on emotional exhaustion and greatly reduces the indirect effect of emotional exhaustion when support is high. In brief, these correlational findings highlight that sustaining helping behaviors during crises relies heavily on both mitigating excessive stress and fostering engagement through tangible organizational support and resource provision. These findings provide actionable psychological evidence for building resilience in community work teams and enhancing emergency management practices.

## Data Availability

The raw data supporting the conclusions of this article will be made available by the authors, without undue reservation.
